# Differences in Peak Impact Accelerations Among Foot Strike Patterns in Recreational Runners

**DOI:** 10.3389/fspor.2022.802019

**Published:** 2022-03-04

**Authors:** Christopher Napier, Lauren Fridman, Paul Blazey, Nicholas Tran, Tom V. Michie, Amy Schneeberg

**Affiliations:** ^1^Centre for Hip Health & Mobility, Vancouver, BC, Canada; ^2^Department of Physical Therapy, University of British Columbia, Vancouver, BC, Canada; ^3^Plantiga Technologies Inc., Vancouver, BC, Canada; ^4^Department of Biomedical Physiology & Kinesiology, Simon Fraser University, Vancouver, BC, Canada; ^5^Independent Researcher, Vancouver, BC, Canada

**Keywords:** running, gait, inertial measurement unit, accelerometer, biomechanics

## Abstract

**Introduction:**

Running-related injuries (RRIs) occur from a combination of training load errors and aberrant biomechanics. Impact loading, measured by peak acceleration, is an important measure of running biomechanics that is related to RRI. Foot strike patterns may moderate the magnitude of impact load in runners. The effect of foot strike pattern on peak acceleration has been measured using tibia-mounted inertial measurement units (IMUs), but not commercially available insole-embedded IMUs. The aim of this study was to compare the peak acceleration signal associated with rearfoot (RFS), midfoot (MFS), and forefoot (FFS) strike patterns when measured with an insole-embedded IMU.

**Materials and Methods:**

Healthy runners ran on a treadmill for 1 min at three different speeds with their habitual foot strike pattern. An insole-embedded IMU was placed inside standardized neutral cushioned shoes to measure the peak resultant, vertical, and anteroposterior accelerations at impact. The Foot strike pattern was determined by two experienced observers and evaluated using high-speed video. Linear effect mixed-effect models were used to quantify the relationship between foot strike pattern and peak resultant, vertical, and anteroposterior acceleration.

**Results:**

A total of 81% of the 187 participants exhibited an RFS pattern. An RFS pattern was associated with a higher peak resultant (0.29 SDs; *p* = 0.029) and vertical (1.19 SD; *p* < 0.001) acceleration when compared with an FFS running pattern, when controlling for speed and limb, respectively. However, an MFS was associated with the highest peak accelerations in the resultant direction (0.91 SD vs. FFS; *p* = 0.002 and 0.17 SD vs. RFS; *p* = 0.091). An FFS pattern was associated with the lowest peak accelerations in both the resultant and vertical directions. An RFS was also associated with a significantly greater peak acceleration in the anteroposterior direction (0.28 SD; *p* = 0.033) than an FFS pattern, while there was no difference between MFS and FFS patterns.

**Conclusion:**

Our findings indicate that runners should be grouped by RFS, MFS, and FFS when comparing peak acceleration, rather than the common practice of grouping MFS and FFS together as non-RFS runners. Future studies should aim to determine the risk of RRI associated with peak accelerations from an insole-embedded IMU to understand whether the small observed differences in this study are clinically meaningful.

## Introduction

Running-related injuries (RRIs) are most often caused by a complex interplay of training load (Hreljac, 2005; Bertelsen et al., 2017; Kalkhoven et al., 2020) and biomechanical movement patterns (Ryan et al., 2006; Napier et al., 2018; Ceyssens et al., 2019). Impact loading, whether measured *via* ground reaction force (GRF) or by peak acceleration, is an important measure of running biomechanics that may be related to RRI (van der Worp et al., 2016; Ceyssens et al., 2019). Foot strike pattern may contribute to the magnitude of impact load in runners, as well as the distribution of this load through anatomical structures (Glauberman and Cavanagh, 2014; Almeida et al., 2015). When measured by GRF, a rearfoot strike (RFS) pattern has been associated with a more prominent impact peak in the vertical direction and a higher vertical loading rate than a forefoot strike (FFS) pattern (Almeida et al., 2015). However, in the horizontal direction, an FFS pattern has been associated with higher peak braking forces (Boyer et al., 2014).

Tibial accelerometers are used regularly in research settings to quantify impact loading during running, with higher peak tibial accelerations having been retrospectively associated with tibial stress fractures (Milner et al., 2006; Pohl et al., 2008). Glauberman and Cavanagh (2014) reported no difference in peak vertical tibial acceleration between RFS and non-RFS runners but found peak resultant and anteroposterior accelerations to be greater among non-RFS runners. These findings are not consistent with those from Gruber et al. (2014) who reported that peak tibial accelerations were significantly greater during RFS than FFS running. The authors of the former study did not specify whether the non-RFS runners ran with a midfoot strike (MFS) or FFS, which might explain these conflicting results. A more recent study compared peak tibial accelerations among RFS, MFS, and FFS runners. Ruder et al. (2019) reported that an MFS pattern more closely resembled an RFS pattern and that both of these patterns exhibited higher peak vertical tibial accelerations than runners who exhibited an FFS pattern.

The popularity of running combined with its high rate of injury has prompted many runners to integrate wearable sensors into their training to monitor and track metrics associated with injury and/or performance. Wearable technologies permit the collection of both biomechanical and training load data, allowing for longitudinal, in-field monitoring of the runner (Napier et al., 2017; Willy, 2018; Moore and Willy, 2019). Quantifying the cumulative stress on the body may inform training program design (e.g., recovery days) and support tissue adaptation (Napier and Willy, 2021). Inertial measurement units (IMUs) are the most pervasive class of wearable sensors on the market, consisting of an accelerometer, gyroscope, and (sometimes) a magnetometer to measure accelerations, angular velocities, and orientation, respectively. These devices are becoming more popular and can be affixed to various locations including the waist, tibia, shoe, and insole.

Inertial measurement units require a consistent and secure mounting to provide a reliable signal for impact-related metrics (Sheerin et al., 2019). The excessive noise that can accompany the acceleration signal because of poor fixation and uncoupling from the body can result in poor correlations between IMU-derived accelerations measured at various locations in addition to GRF metrics (Cheung et al., 2019; Napier et al., 2021). As such, tibia-mounted IMUs are not common among runners outside of research settings owing to the difficulty of consistently and securely fixing them in place at the distal tibia. However, IMUs that are affixed to the shoe or embedded within the insole is gaining in popularity (Napier et al., 2021). Location (proximal to distal), vibration, sampling frequency, dynamic range, and sensor size can influence the magnitude of acceleration reported by an IMU (Norris et al., 2014; Mitschke et al., 2017; Sheerin et al., 2019). Distally mounted IMUs typically provide higher peak acceleration values than those mounted on the tibia, likely due to partial attenuation of the impact by the ankle joint (Giandolini et al., 2014; Cheung et al., 2019; Sheerin et al., 2019). Distally mounted IMUs may also better represent the accelerations experienced by the foot/ankle (Giandolini et al., 2014; Sheerin et al., 2019). This is an important consideration when attempting to quantify the magnitude of impact between different foot strike patterns.

Impact loading (measured using GRF) and peak accelerations from shoe-mounted sensors have typically demonstrated low-to-moderate associations (Cheung et al., 2019; de Pairot, 2020; Napier et al., 2021), but our recent validation study found moderate-to-high associations between impact loading and peak accelerations measured with an insole-embedded IMU (Napier et al., 2021). While poor fixation can lead to increased noise from lace- or wheel-mounted sensors, the location of an insole-embedded sensor has the advantage of being easily and consistently fixated. Furthermore, the location of the sensor provides an opportunity to capture the initial shock of impact at the interface of the foot and the shoe. Peak tibial acceleration occurs after the impact signal has been attenuated by the ankle joint, therefore, measuring it at the foot-shoe interface may yield different findings, especially among different foot strike patterns (Giandolini et al., 2014). In addition to sensor location, running speed (Sinclair et al., 2013a; Boey et al., 2017; Sheerin et al., 2018; Napier et al., 2021) and footwear (Sinclair et al., 2013a,b, 2016; Sinclair and Sant, 2017; Napier et al., 2021) also affect impact-related metrics. Therefore, any investigation with peak acceleration as an outcome must control for these factors.

Commercial and research-grade sensors can be affixed to the waist, tibia, shoe, and insole. The effects of sensor location and foot strike pattern on the magnitude of the peak acceleration need to be understood. While there is currently no established link between RRI and peak accelerations gathered from an insole-embedded IMU, it is important to determine the effect that foot strike pattern may have on the acceleration signal in order to plan and interpret future studies that aim to understand this relationship. The purpose of this study was to compare the peak acceleration signal associated with different foot strike patterns (RFS, MFS, and FFS) when measured with an insole-embedded IMU, while controlling for speed and limb (left vs. right). We hypothesized that an RFS pattern would be associated with higher peak vertical and resultant accelerations and that an FFS pattern would be associated with higher peak anteroposterior accelerations.

## Materials and Methods

### Participants

Healthy runners free of any musculoskeletal or neurological pain who had been running for at least 3 months were recruited from the local running community for a larger study to obtain normative data for insole-embedded IMUs. Participants were excluded if they could not run on a treadmill unaided and if they did not fit the range of shoe sizes available for the study (Men's 8.5–12 US or Women's 6.5–11 US). Participants were screened for inclusion/exclusion criteria *via* an eligibility questionnaire. Written consent was obtained from all participants and ethics approval was granted from the Institutional Clinical Research Ethics Board.

### Experimental Protocol/Procedures

**Figure 1:**
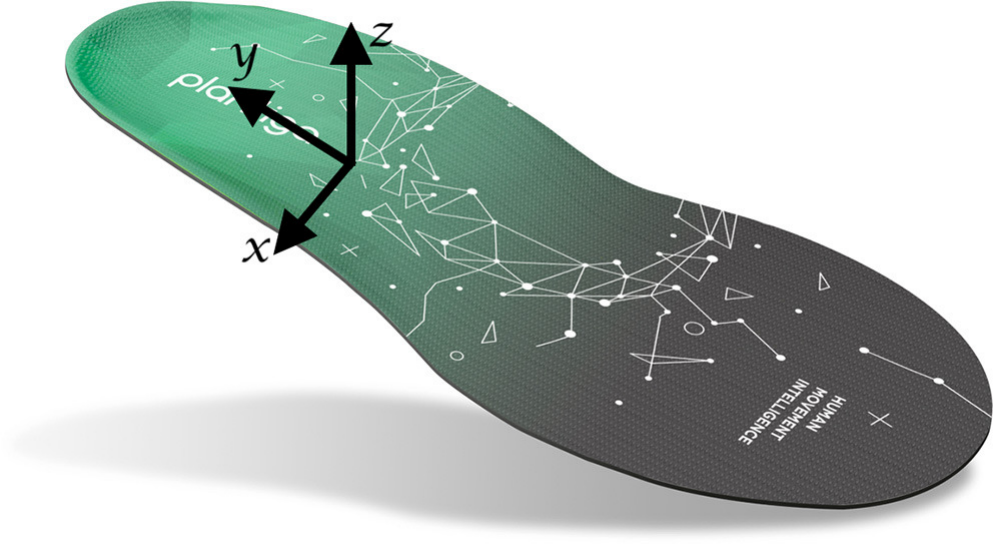
Plantiga insole-embedded inertial measurement unit.

Participants completed an intake questionnaire on arrival and were fitted with a pair of standardized running shoes (Women: New Balance 880v9; Men: New Balance 880v10, New Balance, Boston, USA). Each shoe contained an insole-embedded IMU (Plantiga Technologies Incorporation, Vancouver, Canada; Figure 1). Participants were given 5 min to warm up and familiarize themselves with a motorized treadmill (NordicTrack C700, NordicTrack, Logan, USA). Following the warmup, they ran for 1 min at three different speeds (2.5, 3.0, and 3.5 m/s, in a randomized order) and with their habitual foot strike pattern. Following each trial, the participant was asked if they experienced any pain during the run and if so, to rate it on an 11-point numerical rating scale. Any trial in which a participant reported pain > 2/10 was excluded from the analysis. An iPad (iPad Pro 11, Apple Incorporation, Cupertino, USA) mounted on a tripod was positioned perpendicularly to the treadmill at a distance of 1.65 m from the center so that the sagittal view of the runner could be filmed at 240 frames/s.

### Data Analysis

**Figure 2:**
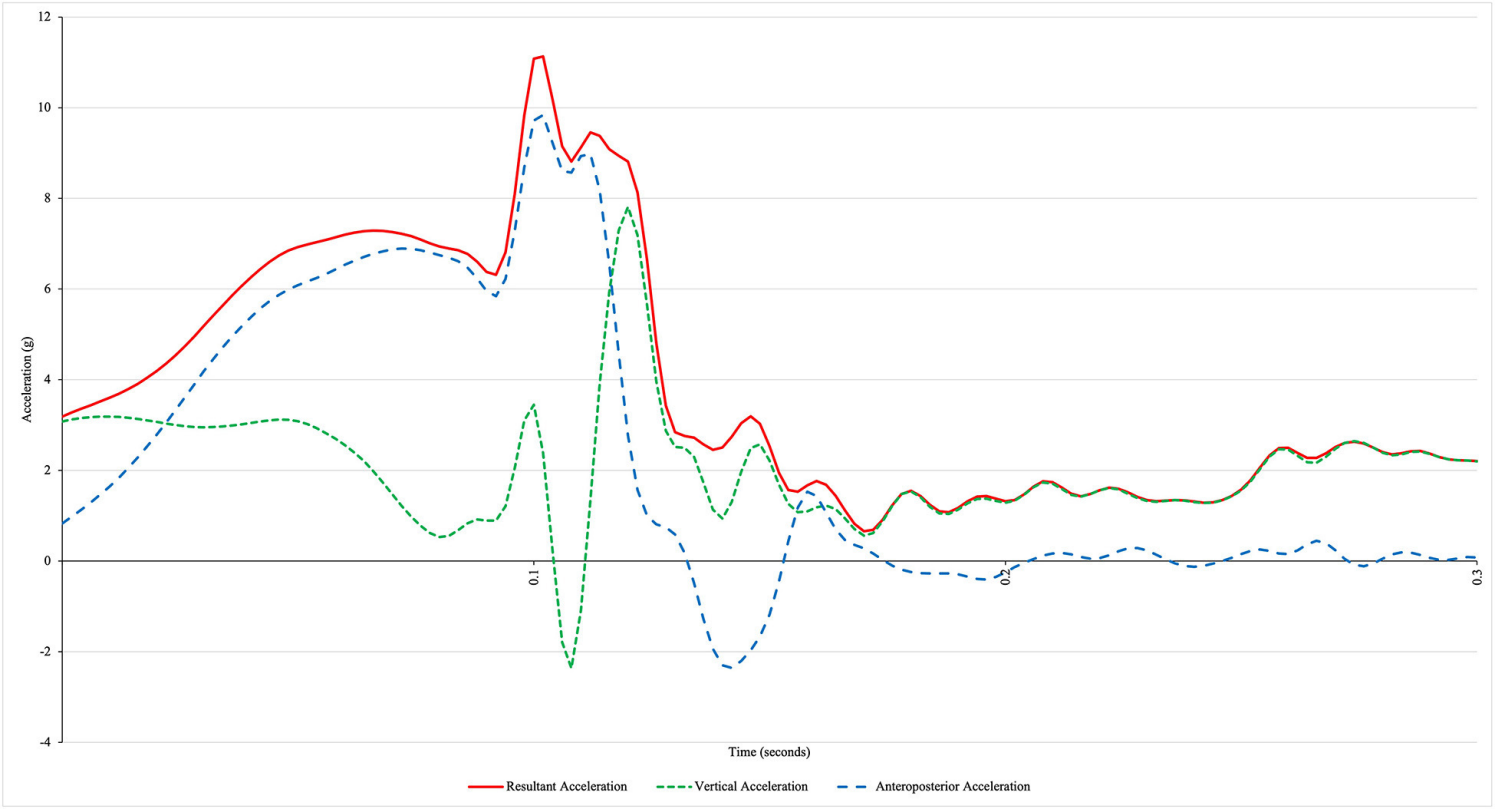
The representative acceleration profile of a rearfoot strike runner using an insole-embedded inertial measurement unit. The x-axis represents the time from initial contact (0 s).

Accelerometer data from the insole-embedded IMU were sampled at 500 Hz. Raw acceleration data were exported from the IMUs into CSV files for signal processing. Discrete variables and accelerometry data were processed using a custom Python code in Python 3.7.6 (Python Software Foundation, Beaverton, USA) for 30 consecutive strides of each foot for each of the three trials. The 30 consecutive strides were taken from the middle of the 1-min data collection window once the participant was up to speed and comfortably running. The orientation of the sensor was such that the vertical axis was perpendicular to the shoe last with the anterior–posterior axis oriented along the heel–toe last of the shoe. As such, the terms vertical and anteroposterior are defined with respect to the shoe/sensor rather than the global coordinate system. The resultant acceleration was calculated using the vertical, anteroposterior, and mediolateral accelerations. Since impact frequencies range from 40 to 60 Hz (Valiant et al., 1987; Winslow and Shorten, 1989), a cut-off frequency of 75Hz was used to ensure that only nonphysiological frequencies were removed from the accelerometry signal (Crowell and Davis, 2011). Accelerometer data from the IMU were filtered via a low-pass, fourth-order Butterworth recursive filter at a cutoff frequency of 75 Hz. Initial contact from accelerometry data was identified at 1 ms before a maximum of the vertical accelerometer signal (Figure 2) (Johnson et al., 2020). Primary outcomes from the accelerometer signal were peak vertical, posterior, and resultant accelerations.

Two physiotherapists (PB and TVM) with experience in clinical gait assessment independently evaluated the foot strike pattern for each foot in each trial, taking into account a randomly chosen set of five continuous strides for each limb from the high-speed video recording. Visual classification of foot strike pattern is a validated method (Esculier et al., 2018) that has been used by others (Hasegawa et al., 2007; Bertelsen et al., 2013) and results in similar agreement with kinematic methods (Altman and Davis, 2012). Foot strike pattern was determined based on the part of the foot that made contact with the treadmill at initial contact (which was visually identified by the observer). Categories were based on Hasegawa et al. (2007) and were divided into 4 types: RFS, MFS, FFS, and mixed foot strike. See Table 1 for definitions. If a participant exhibited more than one type of foot strike on the same limb during the five assessed steps, their foot strike was categorized as being mixed for that limb. Trials with mixed foot strike patterns were excluded from the analysis. Both observers were blinded to participants and running speeds. In cases of disagreement, the two observers met in a consensus meeting.

**Table 1:** Foot strike definitions taken from Hasegawa et al. (2007).

Rearfoot strike (RFS)	RFS was defined as “a footstrike in which the point of the first contact of the foot with the ground was the heel or rear third part of the sole only and in which the midfoot or forefoot portion did not have any contact at footstrike.”
Midfoot strike (MFS)	MFS was defined as “a footstrike in which the point of the first contact of the foot with the ground was not only the rear third of the sole but the midfoot or entire part of the sole.”
Forefoot strike (FFS)	FFS was defined as “a footstrike in which the point of the first contact of the foot with a ground was the forefoot or front half of the sole and in which the heel did not have any contact at the footstrike.”

### Statistical Analysis

The level of agreement on foot strike pattern categorization between observers was determined using percentage agreement and a kappa statistic, between the three categories of RFS, MFS, and FFS. To quantify the relationship between foot strike pattern and the three outcomes of interest (peak resultant, vertical, and anteroposterior acceleration), we used linear mixed-effect models including participant ID as a random effect to account for the correlated nature of the data. Both the crude and adjusted models were examined. Adjusted models included both speed (2.5, 3.0, and 3.5 m/s) and limb (left/right) as they are conceptually important confounders. In addition, the potential for effect modification was explored between foot strike patterns and both the speed and limb with interaction terms. The inclusion of these interaction terms (or not) was determined based on the results of likelihood ratio tests (*p* ≤ 0.05 and interaction was retained). We calculated partially standardized coefficients—by standardizing the outcome variables (peak acceleration) and leaving the categorical variables as is—to allow for interpretability. This makes the parameter estimates directly comparable in terms of SD (Lorah, 2018). All modeling was done in R with the lme4 package (Bates et al., 2015).

## Results

A total of 188 runners met the inclusion criteria and underwent the treadmill running protocol. One participant was excluded based on the absence of a flight phase. As a result, 187 participants (89/98 females/males; age 41.8 ± 12.0 years; body mass index 22.8 ± 2.5 kg/m^2^; running experience 14.9 ± 12.3 years) were included in this study, consisting of 1,122 individual limb-speed trials (187 participants with left and right foot strikes at three different running speeds).

A total of 1,122 video recordings were evaluated by both the observers. Overall, the two observers agreed on 89.7% of all the foot strike patterns and 93.6% of foot strike patterns when mixed foot strike (e.g., RFS/MFS or MFS/FFS) patterns were removed. The kappa values of interobserver agreement on foot strike patterns were 0.75, or “Good,” across all the recordings and 0.81, or “Very Good,” when mixed foot strike patterns were removed (Altman, 1999). After a consensus meeting, both observers agreed on the foot strike patterns for all 1,122 trials. The percentage of foot strike patterns across different speeds are presented in Table 2. The overall rates of RFS, MFS, and FFS were 81.2, 9.8, and 9.0%, respectively.

**Table 2:** Participant foot strike patterns by percentage across running speeds.

	**Running speed**			
	**2.5 m/s**	**3.0 m/s**	**3.5 m/s**	**Overall**
RFS	81.1%	81.6%	83.0%	81.2%
MFS	8.7%	8.4%	8.5%	9.8%
FFS	10.2%	10.1%	8.5%	9.0%

*RFS, rearfoot strike; MFS, midfoot strike; FFS, forefoot strike*.

Before quantifying the relationship between foot strike pattern and the three outcomes of interest (peak resultant, vertical, and anteroposterior acceleration), trials were excluded if the participant rated their pain > 2/10 on an 11-point numerical rating scale (*n* = 8); if the participant displayed a mixed foot strike pattern (*n* = 79); and if the acceleration profile exhibited a double-peak acceleration (*n* = 12), leaving a total of 1,023 individual limb-speed trials to be included in the analysis.

**Figure 3:**
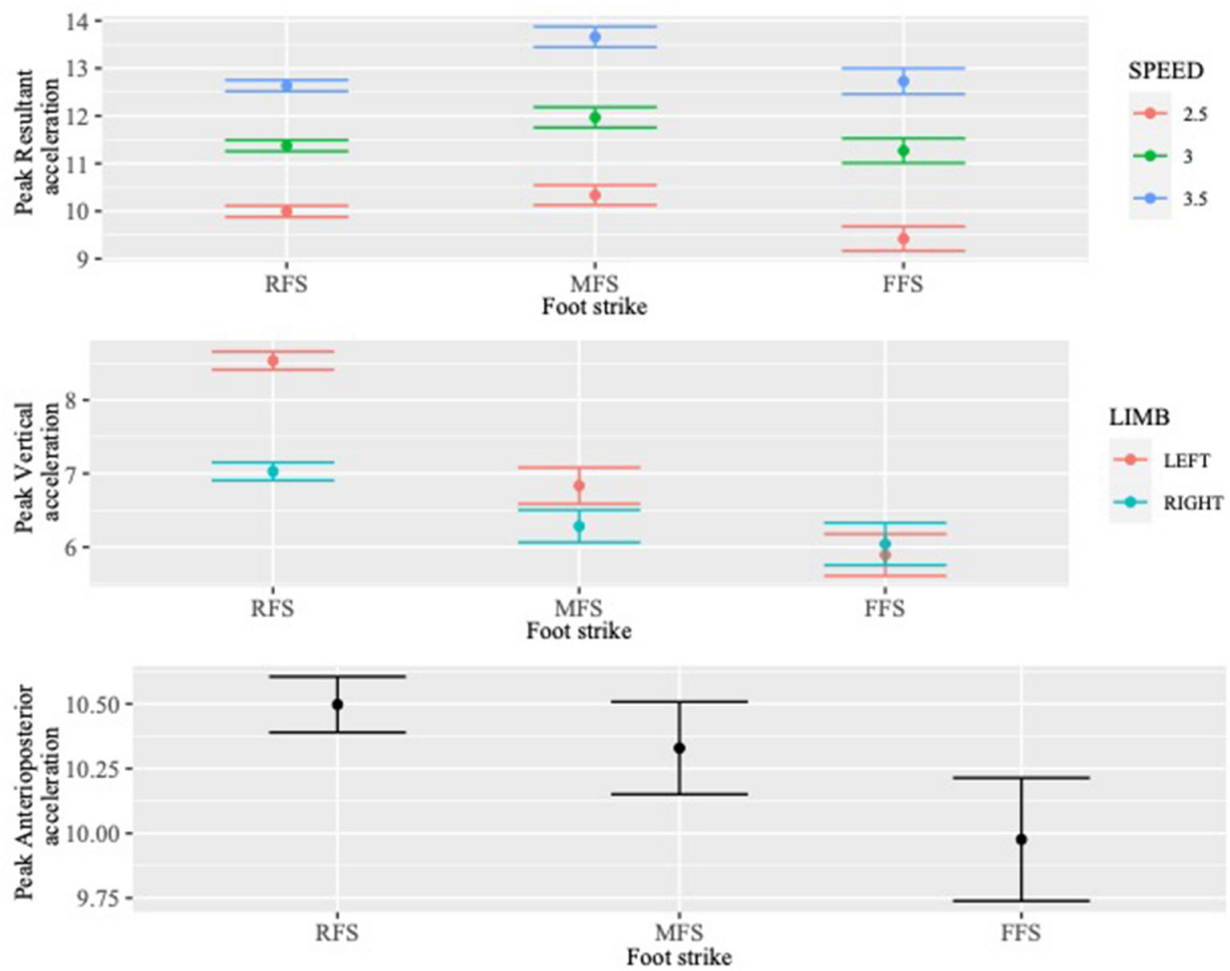
The adjusted relationships between the acceleration outcomes (peak resultant, vertical, and anteroposterior) and foot strike pattern, when controlling for running speed, limb, and interactions as indicated by models (running speed for resultant acceleration and limb for vertical acceleration).

The mean peak resultant, vertical, and anteroposterior accelerations across all speeds and foot strikes were 11.36 ± 1.97, 7.51 ± 2.21, and 10.43 ± 1.87 g, respectively (see Figure 2 for a representative acceleration profile). The final standardized statistical models are presented in Tables 3, 4 (unstandardized models are presented in Supplementary Tables 1, 2). The adjusted relationships between the acceleration outcomes and foot strike pattern, when controlling for running speed, limb, and interactions are shown in Figure 3.

**Table 3:** Final standardized models for the effect of foot strike on peak resultant, vertical, and anteroposterior acceleration controlling for speed and limb.

	**Resultant**			**Vertical**			**Anteroposterior**		
**Predictors**	**Estimates (SD)**	**95% CI**	***p***	**Estimates (SD)**	**95% CI**	***p***	**Estimates (SD)**	**95% CI**	***p***
(Intercept)	−0.76	−0.88 to −0.64	**<0.001**	−0.07	−0.18 to 0.05	0.253	−0.75	−0.87 to −0.63	**<0.001**
Footstrike (MFS)	0.17	−0.03 to 0.37	0.091	−0.77	−0.99 to −0.55	**<0.001**	−0.09	−0.27 to 0.09	0.321
Footstrike (FFS)	−0.29	−0.55 to −0.03	**0.029**	−1.19	−1.46 to −0.93	**<0.001**	−0.28	−0.53 to −0.02	**0.033**
Speed (3.0)	0.70	0.64 to 0.76	**<0.001**	0.52	0.46 to 0.58	**<0.001**	0.72	0.66 to 0.78	**<0.001**
Speed (3.5)	1.34	1.28 to 1.40	**<0.001**	1.06	1.00 to 1.12	**<0.001**	1.31	1.25 to 1.37	**<0.001**
Limb (Right)	0.13	0.08 to 0.17	**<0.001**	−0.68	−0.74 to −0.63	**<0.001**	0.21	0.16 to 0.26	**<0.001**
Footstrike (MFS) * Speed (3.0)	0.13	−0.08 to 0.34	0.225						
Footstrike (FFS) * Speed (3.0)	0.24	0.05 to 0.42	**0.012**						
Footstrike (MFS) * Speed (3.5)	0.35	0.13 to 0.56	**0.001**						
Footstrike (FFS) * Speed (3.5)	0.34	0.15 to 0.53	**0.001**						
Footstrike (MFS) * Limb (Right)				0.43	0.23 to 0.63	**<0.001**			
Footstrike (FFS) * Limb (Right)				0.75	0.57 to 0.93	**<0.001**			
**Random effects**									
σ ^2^		0.13			0.16			0.16	
τ_00_		0.52 _SUBJID_			0.44 _SUBJID_			0.52 _SUBJID_	
ICC		0.8			0.73			0.77	
N		187 _SUBJID_			187 _SUBJID_			187 _SUBJID_	
Observations		1,023			1,023			1,023	
Marginal R^2^/Conditional R^2^		0.345/0.871			0.376/0.829			0.311/0.840	

*Referent categories are rearfoot strike, left limb, and speed of 2.5 m/s*.

*MFS, midfoot strike; FFS, forefoot strike. Significant (p < 0.05) values are in bold*.

**Table 4:** Final standardized models for the effect of foot strike on peak resultant, vertical, and anteroposterior acceleration controlling for speed and limb.

	**Resultant**			**Vertical**			**Anteroposterior**		
**Predictors**	**Estimates (SD)**	**95% CI**	***p***	**Estimates (SD)**	**95% CI**	***p***	**Estimates (SD)**	**95% CI**	***p***
(Intercept)	3.54	3.04 to 4.04	**<0.001**	−1.26	−1.52 to −1.01	**<0.001**	−1.03	−1.28 to −0.77	**<0.001**
Footstrike (MFS)	0.91	0.34 to 1.49	**0.002**	0.43	0.12 to 0.73	**0.006**	0.19	−0.09 to 0.46	0.178
Footstrike (RFS)	0.58	0.06 to 1.09	**0.029**	1.19	0.93 to 1.46	**<0.001**	0.28	0.02 to 0.53	**0.033**
Speed (3.0)	1.85	1.50 to 2.19	**<0.001**	0.07	−0.10 to 0.24	0.435	0.72	0.66 to 0.78	**<0.001**
Speed (3.5)	3.31	2.95 to 3.67	**<0.001**	0.52	0.46 to 0.58	**<0.001**	1.31	1.25 to 1.37	**<0.001**
Limb (Right)	0.25	0.17 to 0.34	**<0.001**	1.06	1.00 to 1.12	**<0.001**	0.21	0.16 to 0.26	**<0.001**
Footstrike (MFS) * Speed (3.0)	−0.21	−0.74 to 0.32	0.429						
Footstrike (RFS) * Speed (3.0)	−0.47	−0.83 to −0.10	**0.012**						
Footstrike (MFS) * Speed (3.5)	0.02	−0.53 to 0.56	0.952						
Footstrike (RFS) * Speed (3.5)	−0.67	−1.05 to −0.29	**0.001**						
Footstrike (MFS) * Limb (RIGHT)				−0.32	−0.57 to −0.06	**0.015**			
Footstrike (RFS) * Limb (RIGHT)				−0.75	−0.93 to −0.57	**<0.001**			
**Random effects**									
σ^2^		0.5			0.16			0.16	
τ_00_		2.03 _SUBJID_			0.44 _SUBJID_			0.52 _SUBJID_	
ICC		0.8			0.73			0.77	
N		187 _SUBJID_			187 _SUBJID_			187 _SUBJID_	
Observations		1,023			1,023			1,023	
Marginal R^2^/Conditional R^2^		0.345/0.871			0.376/0.829			0.311/0.840	

*Referent categories are forefoot strike, left limb, and speed of 2.5 m/s*.

*MFS, midfoot strike; RFS, rearfoot strike. Significant (p < 0.05) values are in bold*.

The final standardized model for peak resultant acceleration included an interaction term between foot strike and speed. Relative to RFS, at 2.5 m/s FFS had a statistically lower mean peak resultant acceleration, estimated to be 0.29 SDs (95% CI: −0.55, −0.03; *p* = 0.029) less than the mean associated with RFS, controlling for limb and speed. An MFS was associated with the highest peak accelerations in the resultant direction (0.91 SD vs. FFS; 95% CI: 0.34, 1.49; *p* = 0.002 and 0.17 SD vs. RFS; 95% CI: −0.03, 0.37; *p* = 0.091), though this result was not statistically significant between RFS and MFS. The results associated with the interaction term indicate that at the faster speeds the difference in peak resultant acceleration between RFS and FFS decreased statistically significantly (3.0 m/s: 0.24 SD; *p* = 0.012 and 3.5 m/s: 0.34 SD; *p* = 0.001), while the difference between RFS and MFS only increased at the 3.5 m/s speed (0.35 SD; *p* = 0.001).

The final model for peak vertical acceleration included an interaction term between foot strike and limb. Both MFS and FFS had statistically lower means for peak vertical acceleration relative to RFS on the left limb, estimated to be 0.77 SD (95% CI: −0.99, −0.55; *p* < 0.001) and 1.19 SD (95% CI: −1.46, −0.93; *p* < 0.001) lower than the mean associated with RFS, respectively, controlling for limb and speed. The results associated with the interaction term indicate that differences between foot strike patterns were smaller on the right limb than on the left limb. Specifically, the difference in peak vertical acceleration decreased statistically significantly between RFS and MFS (0.43 SD; *p* < 0.001) and between RFS and FFS (0.75 SD; *p* < 0.001), respectively, for the right limb.

The final model for peak anteroposterior acceleration did not include any interaction terms between foot strike and speed or limb. Relative to RFS, FFS had a statistically lower mean for peak anteroposterior acceleration, estimated to be 0.28 SD (95% CI: −0.53, −0.02; *p* = 0.033) lower than the mean associated with RFS, controlling for limb and speed. Relative to RFS, MFS did not have a statistically different mean for peak anteroposterior acceleration.

## Discussion

The purpose of this investigation was to compare the peak acceleration signal associated with different foot strike patterns (RFS, MFS, and FFS) when measured with an insole-embedded inertial measurement unit (IMU) while controlling for speed and limb. We hypothesized that an RFS pattern would be associated with higher peak vertical and resultant accelerations and an FFS pattern would be associated with higher peak anteroposterior accelerations.

In support of our hypotheses, an RFS pattern was associated with a higher peak resultant and vertical acceleration when compared with an FFS running pattern. However, an MFS was associated with the highest peak accelerations in the resultant direction. An FFS pattern was associated with the lowest peak accelerations in both the resultant and vertical directions. In the anteroposterior direction, our hypothesis was not supported. An RFS was associated with a significantly greater peak acceleration than an FFS pattern, while there was no difference between MFS and FFS patterns.

Studies often group foot strike patterns into RFS and non-RFS based on an assumption that MFS and FFS runners exhibit lower impact loading than RFS runners. This practice is also done out of expedience because RFS runners often make up more than 80% of all runners with MFS and FFS making up the remainder. In a study comparing RFS to non-RFS runners, Glauberman and Cavanagh (2014) reported no difference in peak vertical tibial acceleration, but found peak resultant and anteroposterior accelerations to be greater among non-RFS runners. It is not known what proportion of the non-RFS runners exhibited an MFS vs. an FFS, which might have masked a difference between these categories. Our findings align with those of Ruder et al. (2019) who reported higher peak vertical tibial acceleration values in RFS and MFS runners compared to FFS runners. Peak resultant and anteroposterior accelerations were not reported. Our results do not align with the findings of Boyer et al. (2014) and Glauberman and Cavanagh (2014) both of whom reported higher impact loading in the anteroposterior direction in FFS runners. While we acknowledge that Boyer et al. used GRF metrics (aligned to the global coordinate system) and Ruder et al. and Glauberman et al. used accelerometers mounted on the tibia rather than embedded in the insole, in our study participants who ran with an FFS had the lowest peak anteroposterior accelerations with RFS runners exhibiting the highest. Our findings, taken with the results from Boyer et al. (2014) and Ruder et al. (2019) further challenge the standard practice of grouping MFS and FFS runners together when making comparisons regarding impact loading. However, since the acceleration properties are specific to the body segment on which the accelerometer is fixated, and thus depend on the device placement, the location of the device should be carefully considered when setting up an experiment and writing the research question.

While there were statistically significant differences between foot strike patterns, the absolute differences between foot strike patterns were minimal in the resultant and anteroposterior directions. These differences decreased for RFS runners in the resultant direction as speed increased. The largest differences were in the vertical direction, where RFS runners exhibited peak accelerations an average of 2.94 g greater than FFS runners, controlling for speed and limb. The orientation of the vertical axis of the sensor may have contributed to this difference as it would have more closely aligned with the resultant direction in the RFS runners and would have been offset in the FFS runners. One caveat when interpreting our results is the orientation of the insole-embedded sensors, which can vary depending on the foot strike pattern. The reference frame for these sensors is aligned with the shoe as opposed to the ground (as when studying the GRF signal). Tibial accelerometers also align the vertical axis with the tibia, but this orientation does not vary as much between different foot strike patterns. The resultant signal is a more robust metric as it takes into account all axes and is independent of accelerometer alignment (Sheerin et al., 2019). However, it should be noted that while peak vertical tibial acceleration has been associated with RRI, at this time there are no studies that have examined the relationship between RRIs and peak acceleration in any direction using insole-embedded IMUs.

One notable finding was the effect that limb had on the peak vertical acceleration outcome. While impact asymmetry was not an outcome of this study, it is worth discussing briefly here given this interesting result. Specifically, the left limb exhibited an average peak vertical acceleration that was 0.68 standard deviations greater than the right limb when controlling for speed. This limb effect was greatest in RFS runners and because they make up >80% of all participants this translated to an overall effect. It is possible that an FFS pattern is associated with a more symmetrical gait, though we are not aware of any studies that have investigated this question. Gait symmetry is often assumed for simplicity of data collection and analysis, with many studies relying only on unilateral data collection, but the evidence is far from clear that this is the case (Sadeghi et al., 2000). Previous studies have indicated that separate limbs are preferentially used by individuals for stabilization, propulsion, or braking during walking gait, and it can be assumed that this is also the case in running (Sadeghi et al., 2000; Potdevin et al., 2008; Seeley et al., 2008). One factor that can influence gait symmetry is a limb length discrepancy (Kaufman et al., 1996). While we did not measure limb lengths, it is unlikely that limb length discrepancies would be asymmetrically distributed to this degree in our sample. Limb dominance has also been proposed as a potential reason for asymmetry. However, Hamill et al. (1984) did not find any statistically significant differences between the dominant and non-dominant limb when ground reaction force parameters were compared during overground running. Perhaps the most likely reason for this finding is previous injury history. Our inclusion criteria specified that participants must be “free of any musculoskeletal or neurological pain” and we excluded any trials in which a participant reported pain > 2/10. However, despite being currently pain-free, previous injury history could have influenced the symmetry of their gait. Previous lower extremity overuse injuries can demonstrate significant differences between the injured and uninjured limbs (Zifchock et al., 2006). Radzak et al. (2017) also found significant GRF asymmetries among a group of healthy runners. These previous injuries might not be randomly distributed between limbs, and perhaps this is where limb dominance has an effect. One final possibility may be related to the physical set up of the research space. The treadmill was bordered on the left side by a wall and the right side by open space. This may have had a perceptual influence on the participants as they ran, causing them to land harder on their left side than their right because of the safety of having a wall next to them. Certainly, this finding warrants further investigation, but at this point, we are not able to answer this question with the data we have collected.

Given the known effect of footwear and speed on peak accelerations during running (Napier et al., 2021), a strength of this study was that we used a standard neutral cushioned shoe for all participants and collected data at three fixed speeds, presented in a randomized order. Our statistical models also controlled for both speed and limb. Speed moderated the effect of the foot strike pattern on the peak resultant acceleration. The mean difference between RFS and FFS decreased as running speed increased, whereas the relationship between MFS and FFS did not change. Speed also did not affect the relationship between foot strike patterns for peak vertical or anteroposterior acceleration. Overall, speed had a greater effect than foot strike pattern on all three acceleration outcomes, with a significant increase in peak resultant, vertical, and anteroposterior accelerations as running speed increased. The increase in peak vertical acceleration from 2.5 to 3.5 m/s, when controlling for limb and foot strike, was comparable with the difference between RFS and FFS runners, controlling for limb and speed, but for peak resultant and anteroposterior acceleration the increase from 2.5 to 3.5 m/s was three to seven times greater than the difference between RFS/MFS and FFS. The clinical implications of this finding are that decreasing running speed may have a larger effect on impact loading than changing foot strike patterns. This agrees with our previous findings that speed has a greater effect on reducing vertical and posterior GRF outcomes than does foot strike angle.

Another strength of this study was that we collected running data while participants exhibited their habitual foot strike patterns, as recommended by Boyer et al. (2014). The distribution of foot strike patterns in this study were comparable to previous studies (Hasegawa et al., 2007; Larson et al., 2011; Ruder et al., 2019), with the large majority exhibiting an RFS pattern. Overall mean values for peak vertical acceleration in this study were comparable to our previous study and increased with increasing running speed (Napier et al., 2021). Peak resultant and anteroposterior accelerations were not calculated in our previous study and there are no other known reported values for an insole-embedded IMU. Given that different findings might be observed depending on sensor location, future studies may wish to choose a sensor location based on the anatomical structure being investigated. This study is an important step forward to establish normative data for peak accelerations in multiple directions measured by an insole-embedded IMU.

There are several limitations of this study. First, there is currently unknown clinical meaningfulness or risk quantification of the peak accelerations gathered from an insole-embedded IMU. Future longitudinal studies should seek to understand the relationship between RRI and peak acceleration from an insole-embedded IMU. Second, we did not measure kinematics so we do not know what other variables (lower limb geometry, step rate/length, etc.) could have contributed to some of the variances between foot strike patterns. Third, while controlling for footwear adds strength to our results, it limits generalizability to other footwear types such as minimalist or maximalist running shoes (Napier et al., 2021). Finally, data were collected on a treadmill in this study and results could be different when running overground.

## Conclusion

Our findings suggest that runners should be grouped by RFS, MFS, and FFS when making comparisons regarding impact loading, rather than the common practice of grouping MFS and FFS together as non-RFS runners. While at this time there are no studies that have examined the relationship between RRI and peak acceleration using an insole-embedded IMU, this study is an important first step to compare impact-related characteristics of runners who exhibit different habitual foot strike patterns. Our findings also suggest that speed may be a more important factor than foot strike pattern when it comes to the magnitude of peak impact acceleration. Future longitudinal studies to determine the risk of RRI associated with peak accelerations from an insole-embedded IMU are needed to determine whether peak accelerations measured with an insole-embedded IMU can help identify individuals at higher injury risk and whether the small observed differences in this study are clinically meaningful.

## Data Availability Statement

The raw data supporting the conclusions of this article will be made available by the authors, without undue reservation.

## Ethics Statement

The studies involving human participants were reviewed and approved by University of British Columbia Clinical Research Ethics Board. Written informed consent from the participants' legal guardian/next of kin was not required to participate in this study in accordance with the national legislation and the institutional requirements.

## Author Contributions

CN designed the study and drafted the initial manuscript. LF and NT carried out all data collection. PB and TM rated foot strike patterns among participants. CN, LF, and AS were involved in data analysis. CN, LF, NT, PB, TM, and AS reviewed and edited the final manuscript. All the authors were involved in the interpretation and discussion of the results. All authors contributed to the article and approved the submitted version.

## Funding

CN is supported by the Michael Smith Foundation for Health Research/Plantiga Health Professional-Investigator Award (grant number: HPI-2020-0719).

## Conflict of Interest

LF and NT are employees of Plantiga Technologies, Inc. The remaining authors declare that the research was conducted in the absence of any commercial or financial relationships that could be construed as a potential conflict of interest.

## Publisher's Note

All claims expressed in this article are solely those of the authors and do not necessarily represent those of their affiliated organizations, or those of the publisher, the editors and the reviewers. Any product that may be evaluated in this article, or claim that may be made by its manufacturer, is not guaranteed or endorsed by the publisher.
