# Supplementary material for: Differences in Peak Impact Accelerations Among Foot Strike Patterns in Recreational Runners
Source: Front Sports Act Living. 2022 Mar 4;4:802019. doi: 10.3389/fspor.2022.802019 (PMC8931222; doi:10.3389/fspor.2022.802019)
Supplement: Supplementary file 1 [file Table_1.docx]

# Supplementary Files

**Supplementary Table 1.**  Final unstandardized models for peak resultant, vertical, and anteroposterior acceleration controlling for footstrike, speed and limb. Referent categories are rearfoot strike, left limb and speed of 2.5 m/s. Significant (p<0.05) values are in bold.

|  | **RESULTANT (g)** | | | **VERTICAL (g)** | | | **ANTEROPOSTERIOR (g)** | | | |  |
| --- | --- | --- | --- | --- | --- | --- | --- | --- | --- | --- | --- |
| *Predictors* | *Estimates* | *95% CI* | *p* | *Estimates* | *95% CI* | *p* | *Estimates* | *95% CI* | | *p* | |
| (Intercept) | 9.87 | 9.64 – 10.11 | **<0.001** | 7.36 | 7.11 – 7.62 | **<0.001** | 9.03 | 8.80 – 9.25 | | **<0.001** | |
| FOOTSTRIKE [MFS] | 0.34 | -0.05 – 0.73 | 0.091 | -1.70 | -2.18 – -1.22 | **<0.001** | -0.17 | -0.50 – 0.16 | | 0.321 | |
| FOOTSTRIKE [FFS] | -0.58 | -1.09 – -0.06 | **0.029** | -2.64 | -3.22 – -2.06 | **<0.001** | -0.52 | -1.00 – -0.04 | | **0.033** | |
| SPEED [3.0] | 1.38 | 1.26 – 1.50 | **<0.001** | -1.51 | -1.63 – -1.38 | **<0.001** | 1.34 | 1.23 – 1.45 | | **<0.001** | |
| SPEED [3.5] | 2.64 | 2.52 – 2.76 | **<0.001** | -1.15 | 1.01 – 1.29 | **<0.001** | 2.46 | 2.34 – 2.57 | | **<0.001** | |
| LIMB [RIGHT] | 0.25 | 0.17 – 0.34 | **<0.001** | 2.34 | 2.20 – 2.48 | **<0.001** | 0.39 | 0.30 – 0.48 | | **<0.001** | |
| FOOTSTRIKE [MFS] * SPEED[3.0] | 0.26 | -0.16 – 0.67 | 0.225 |  |  |  |  |  | |  | |
| FOOTSTRIKE [FFS] * SPEED[3.0] | 0.47 | 0.10 – 0.83 | **0.012** |  |  |  |  |  | |  | |
| FOOTSTRIKE [MFS] * SPEED[3.5] | 0.69 | 0.27 – 1.11 | **0.001** |  |  |  |  |  | |  | |
| FOOTSTRIKE [FFS] * SPEED[3.5] | 0.67 | 0.29 – 1.05 | **0.001** |  |  |  |  |  | |  | |
| FOOTSTRIKE [MFS] * LIMB[RIGHT] |  |  |  | 0.96 | 0.52 – 1.40 | **<0.001** |  |  | |  | |
| FOOTSTRIKE [FFS] * LIMB[RIGHT] |  |  |  | 1.66 | 1.26 – 2.05 | **<0.001** |  |  | |  | |
| **Random Effects** |  |  |  |  |  |  |  |  |  | |  |
| σ^2^ | 0.5 | |  | 0.81 | |  | 0.55 | |  | |  |
| τ_00_ | 2.03 _SUBJID_ | |  | 2.14 _SUBJID_ | |  | 1.82 _SUBJID_ | |  | |  |
| ICC | 0.8 | |  | 0.73 | |  | 0.77 | |  | |  |
| N | 187 _SUBJID_ | |  | 187 _SUBJID_ | |  | 187 _SUBJID_ | |  | |  |
| Observations | 1023 | |  | 1023 | |  | 1023 | |  | |  |
| Marginal R^2^ / Conditional R^2^ | 0.345 / 0.871 | |  | 0.376 / 0.829 | |  | 0.311 / 0.840 | |  | |  |

MFS, midfoot strike; FFS, forefoot strike; g, gravitational forces.

**Supplementary Table 2.**  Final unstandardized models for peak resultant, vertical, and anteroposterior acceleration controlling for footstrike, speed and limb. Referent categories are forefoot strike, left limb and speed of 2.5 m/s. Significant (p<0.05) values are in bold.

|  | **RESULTANT (g)** | | | **VERTICAL (g)** | | | **ANTEROPOSTERIOR (g)** | | |
| --- | --- | --- | --- | --- | --- | --- | --- | --- | --- |
| *Predictors* | *Estimates* | *95% CI* | *p* | *Estimates* | *95% CI* | *p* | *Estimates* | *95% CI* | *p* |
| (Intercept) | 9.3 | 8.79 – 9.80 | **<0.001** | 4.72 | 4.16 – 5.28 | **<0.001** | 8.51 | 8.04 – 8.98 | **<0.001** |
| FOOTSTRIKE [MFS] | 0.91 | 0.34 – 1.49 | **0.002** | 0.94 | 0.27 – 1.61 | **0.006** | 0.35 | -0.16 – 0.87 | 0.178 |
| FOOTSTRIKE [RFS] | 0.58 | 0.06 – 1.09 | **0.029** | 2.64 | 2.06 – 3.22 | **<0.001** | 0.52 | 0.04 – 1.00 | **0.033** |
| SPEED [3.0] | 1.85 | 1.50 – 2.19 | **<0.001** | 1.15 | 1.01 – 1.29 | **<0.001** | 1.34 | 1.23 – 1.45 | **<0.001** |
| SPEED [3.5] | 3.31 | 2.95 – 3.67 | **<0.001** | 2.34 | 2.20 – 2.48 | **<0.001** | 2.46 | 2.34 – 2.57 | **<0.001** |
| LIMB [RIGHT] | 0.25 | 0.17 – 0.34 | **<0.001** | 0.15 | -0.22 – 0.52 | 0.435 | 0.39 | 0.30 – 0.48 | **<0.001** |
| FOOTSTRIKE [MFS] * SPEED[3.0] | -0.21 | -0.74 – 0.32 | 0.429 |  |  |  |  |  |  |
| FOOTSTRIKE [RFS] * SPEED[3.0] | -0.47 | -0.83 – -0.10 | **0.012** |  |  |  |  |  |  |
| FOOTSTRIKE [MFS] * SPEED[3.5] | 0.02 | -0.53 – 0.56 | 0.952 |  |  |  |  |  |  |
| FOOTSTRIKE [RFS] * SPEED[3.5] | -0.67 | -1.05 – -0.29 | **0.001** |  |  |  |  |  |  |
| FOOTSTRIKE [MFS] * LIMB[RIGHT] |  |  |  | -0.7 | -1.26 – -0.14 | **0.015** |  |  |  |
| FOOTSTRIKE [RFS] * LIMB[RIGHT] |  |  |  | -1.66 | -2.05 – -1.26 | **<0.001** |  |  |  |
| **Random Effects** |  |  |  |  |  |  |  |  |  |
| σ^2^ | 0.5 | |  | 0.81 | |  | 0.55 | |  |
| τ_00_ | 2.03 _SUBJID_ | |  | 2.14 _SUBJID_ | |  | 1.82 _SUBJID_ | |  |
| ICC | 0.8 | |  | 0.73 | |  | 0.77 | |  |
| N | 187 _SUBJID_ | |  | 187 _SUBJID_ | |  | 187 _SUBJID_ | |  |
| Observations | 1023 | |  | 1023 | |  | 1023 | |  |
| Marginal R^2^ / Conditional R^2^ | 0.345 / 0.871 | |  | 0.376 / 0.829 | |  | 0.311 / 0.840 | |  |

MFS, midfoot strike; RFS, rearfoot strike; g, gravitational forces.
